# Fire Performance, Thermal Degradation and Combustion Products of Flame-Retardant Hybrid Epoxy Composites

**DOI:** 10.3390/ma19214754

**Published:** 2026-11-09

**Authors:** Kamila Mizera, Kamila Sałasińska

**Affiliations:** 1 Central Institute for Labour Protection— National Research Institute (CIOP-PIB), Czerniakowska St. 16, 00-701 Warsaw, Poland; kamila.salasinska@pw.edu.pl; 2 Faculty of Materials Science and Engineering, Warsaw University of Technology, Wołoska 141, 02-507 Warsaw, Poland

**Keywords:** hybrid epoxy composites, flame retardancy, fire behavior, thermal degradation, combustion products

## Abstract

**Highlights:**

- ACMGBHF exhibited the longest ignition time (206 s) and the lowest pHRR (79 kW·m^−2^).
- ACGBF achieved the lowest average HRR (8 kW·m^−2^) and THR (4 MJ·m^−2^).
- VACMGBHF showed the lowest degradation rate (2.03%·min^−1^).
- Hybrid architectures significantly influenced smoke generation and char formation.
- STA/FT-IR and Purser furnace analyses characterized evolved thermal degradation gases.

**Abstract:**

The fire performance of hybrid epoxy composites depends on both the flame-retardant system and the reinforcement architecture. This study investigates the effects of different combinations of synthetic and natural fabrics and functional fillers on the fire behavior, thermal degradation, smoke production, and combustion products of epoxy composites containing 10 wt.% expandable graphite (EG) and 10 wt.% ammonium polyphosphate (APP). A glass-fiber-reinforced composite was used as the reference. The materials were characterized by cone calorimetry, smoke density measurements, horizontal burning tests, thermogravimetric analysis (TG/DTG), differential scanning calorimetry (DSC), simultaneous thermal analysis coupled with Fourier-transform infrared spectroscopy (STA/FT-IR), and combustion-effluent analysis using a Purser furnace coupled with a Gasmet DX4000 gas analyzer. Significant differences in fire performance were observed among the formulations. VACMGBHF exhibited the longest time to ignition (206 s), the lowest peak heat release rate (79 kW·m^−2^), and the lowest maximum mass-loss rate during the principal thermo-oxidative degradation stage (2.03%·min^−1^). It also retained 38.8% of its initial mass at 900 °C, the highest value among the hybrid composites. In contrast, ACGBF showed the lowest average heat release rate (8 kW·m^−2^), maximum average rate of heat emission (20 kW·m^−2^), and total heat release (4 MJ·m^−2^). Smoke production also depended on composite composition. The lowest maximum specific optical density was recorded for the glass-fabric reference (42), whereas ACGBF exhibited the lowest total smoke release (123 m^2^·m^−2^). Visual examination revealed differences in the continuity and morphology of the post-combustion residues, with several hybrid formulations forming expanded, cohesive carbonaceous–mineral layers. TG/DTG, DSC, and STA/FT-IR analyses further demonstrated formulation-dependent degradation behavior and evolved-gas profiles. All composites achieved the HB classification in the horizontal burning test. The findings demonstrate that reinforcement architecture and filler composition influence heat release, smoke generation, and thermal stability in different ways, highlighting the need to evaluate these parameters together when designing flame-retardant epoxy composites.

## 1. Introduction

The development of advanced polymer composites has attracted increasing attention due to the growing demand for lightweight, mechanically efficient and environmentally sustainable materials. Fiber-reinforced polymer composites, including glass- and carbon-fiber-reinforced polymers, offer high specific stiffness and strength, design flexibility and the potential to reduce structural weight and material consumption [1,2]. However, despite these advantages, the application of polymer composites in areas requiring enhanced fire safety remains challenging because many polymer matrices are combustible and can release substantial amounts of heat, smoke and potentially toxic combustion products when exposed to fire. Consequently, the development of composite materials with reduced flammability and smoke emissions has become an important research objective, particularly where fire conditions may threaten human health and safety [3,4,5].

Hybrid polymer composites represent a promising approach to tailoring material properties through the combination of two or more types of reinforcing fibers within a common polymer matrix. This approach makes it possible to combine the complementary characteristics of different reinforcements and adjust the balance between mechanical performance, weight and production cost. For example, combining high-performance carbon fibers with less expensive glass fibers allows the properties and cost of a composite to be adjusted by varying the fiber ratio and reinforcement architecture [6,7]. Depending on the intended properties, hybrid composites can incorporate different fiber types in successive layers, within the same layer or in hybrid textile structures. Common reinforcements include glass, carbon, aramid and basalt fibers, as well as natural fibers such as flax, hemp and sisal [8,9]. The combination of natural and synthetic reinforcements is of particular interest because it offers opportunities to reduce dependence on conventional synthetic materials while maintaining the required functional properties. In addition, the use of bio-based constituents and waste-derived fillers may contribute to more sustainable material solutions and improved resource efficiency [8].

The properties of hybrid composites depend on several interacting factors, including the type and proportion of the reinforcing fibers, fiber orientation, stacking sequence, matrix characteristics, fiber–matrix interfacial adhesion and manufacturing conditions [6,10]. Previous studies have demonstrated that the selection and arrangement of reinforcing layers can substantially influence the mechanical performance of hybrid composites. Zachariah et al. [11] investigated carbon–aramid–epoxy composites with different stacking sequences and demonstrated the importance of reinforcement arrangement for impact resistance. Similarly, studies examining hybrid epoxy composites containing carbon and aramid fabrics have shown that the number, position and orientation of reinforcing layers affect impact response and energy absorption [12]. Other investigations have explored the modification of fiber–matrix interfaces to improve composite performance. For example, the incorporation of graphene oxide into glass-fiber-reinforced epoxy composites improved interfacial compatibility and shear strength [13]. Matykiewicz and Barczewski [14] investigated hybrid epoxy composites reinforced with flax and modified basalt fibers, demonstrating the potential for incorporating natural-fiber layers without adversely affecting selected mechanical properties. These studies illustrate the flexibility of hybrid composite design, while also showing that the properties of the final material depend on the interactions between its constituents and cannot be predicted solely from the characteristics of the individual components.

In addition to mechanical performance, the thermal stability and fire behavior of polymer composites are critical considerations in their development. Natural fibers, although attractive from a sustainability perspective, contain lignocellulosic components that undergo thermal degradation at relatively low temperatures and may adversely affect the fire performance of polymer composites [15,16]. Furthermore, the polymer matrix and other organic constituents can contribute to flame propagation, heat release and smoke formation. Consequently, fire behavior is governed by the combined effects of the matrix, reinforcing materials, additives, constituent proportions and composite architecture. Increasing the content of inorganic or thermally stable components does not necessarily ensure improved fire performance, since the interactions between the constituents and their decomposition behavior can significantly influence the combustion process [4,5].

To address these challenges, research has increasingly focused on incorporating flame retardants and functional fillers into polymer composites. Flame-retardant systems can inhibit combustion through different mechanisms, including gas-phase radical reactions, endothermic decomposition and the formation of protective layers that restrict heat and mass transfer. Inorganic mineral fillers can complement these mechanisms by promoting the formation and stabilization of a condensed protective phase during thermal decomposition. In particular, combinations of flame retardants and mineral fillers have demonstrated potential for reducing heat release and modifying smoke generation [17]. Vermiculite-based systems have also attracted interest because of their potential to form thermally stable structures that act as barriers to heat and volatile combustion products [18]. However, the effectiveness of these systems depends on filler composition, concentration, dispersion and interactions with the polymer matrix and reinforcing constituents. Moreover, improvements in flame retardancy do not necessarily result in proportional reductions in smoke emissions or toxic combustion products, which therefore require separate evaluation [4,5].

The incorporation of particulate fillers derived from renewable resources or waste materials provides an additional opportunity to develop more sustainable hybrid composites. Ground hazelnut shells, for example, represent a bio-based material that can be considered a particulate constituent in polymer composite formulations. Their incorporation alongside mineral fillers and conventional reinforcing fabrics may offer a means of combining material utilization with the modification of composite properties. However, the thermal decomposition of lignocellulosic fillers and their interactions with the polymer matrix and flame-retardant system require careful consideration. Their effects on flammability, smoke emissions and the composition of combustion products must therefore be established experimentally rather than assumed to be beneficial.

Although substantial research has been devoted to hybrid fiber-reinforced composites, flame-retardant polymer systems and functional mineral fillers, the combined influence of diverse reinforcement architectures, particulate constituents and flame-retardant formulations on flammability, smoke emissions and toxic combustion products remains an important area for further investigation. In particular, the assessment of hybrid systems incorporating different combinations of flax, aramid, basalt, carbon and glass fabrics together with vermiculite, glass microspheres and ground hazelnut shells may provide further insight into the relationships between composite composition and fire behavior. A comprehensive evaluation of these characteristics is necessary because the contribution of individual constituents cannot be directly extrapolated to the performance of the complete hybrid material.

Although hybrid fiber-reinforced composites, flame-retardant polymer systems and mineral fillers have been widely studied [19,20,21], their combined effects on fire behavior are still not fully understood [20]. This is particularly relevant to composites containing different types of reinforcing fabrics, such as flax, aramid, basalt, carbon and glass, combined with vermiculite, glass microspheres and ground hazelnut shells. These components may interact during thermal degradation and combustion. Their combined effects on flammability, smoke emissions, toxic gases and residue formation therefore require further investigation [22].

In this context, the present study investigates the fire-related properties of patented hybrid polymer composites covered by Polish patent PL 248017 B1 [23]. The investigated materials incorporate different arrangements of flax, aramid, basalt, carbon and glass fabrics, particulate fillers comprising vermiculite, glass microspheres and ground hazelnut shells, and a flame-retardant system based on commercially available powdered chemical compounds incorporated into the polymer resin. The novelty of the investigated approach lies in the integration of these different reinforcement types and functional particulate constituents within a patented hybrid composite system, providing a basis for examining their combined effects on material flammability, smoke emissions and toxic combustion products. Rather than considering the contributions of individual fibers or flame-retardant additives in isolation, the study focuses on the fire behavior of the resulting composite formulations and the influence of their constituent selection and reinforcement architecture. The findings are intended to improve understanding of the relationship between hybrid composite design and fire-related performance and to assess the potential of the patented material concept for the development of composites with reduced flammability and smoke emissions.

## 2. Materials and Methods

### 2.1. Materials

The epoxy resin RenLam LY 113 (viscosity: 580 mPa·s at 25 °C; density: 1.16 g·cm^−3^) and the curing agent Ren HY 97-1 (viscosity: 20 mPa·s at 25 °C; density: 0.95 g·cm^−3^), supplied by Huntsman Advanced Materials GmbH (Basel, Switzerland), were used as the polymer matrix. The resin and curing agent were mixed at a mass ratio of 100:30.

The following fabrics were used to prepare the composites: Biax 400 g·m^−2^ carbon fabric (C; actual areal weight: 410 g·m^−2^) from Saertex (Saerbeck, Germany); aramid fabric (A; areal weight: 110 g·m^−2^) from P.P.H.U. Surfpol (Rawa Mazowiecka, Poland); BIAX 400 g·m^−2^ glass fabric (G; actual areal weight: 411 g·m^−2^) from Saertex (Saerbeck, Germany); Basfiber^®^ BT11/1 basalt fabric (B; areal weight: 380 g·m^−2^) from Stone Vek (Moscow, Russia); and flax fabric (F; areal weight: 500 g·m^−2^) from Safilin (Sailly-sur-la-Lys, France). Three powdered fillers were used to prepare the hybrid composites.

Superfine raw vermiculite (V) with grain size in the range of 0.3–1 mm (at least 80%) from Vermeko Sp. z.o.o. (Lublin, Poland) was added at 25.5 wt.% to the appropriate, previously deducted amount of resin with curing agent. CO_2_-filled glass beads (M) with a diameter of 30–115 μm, with the trade name Mikrobalon DT-99, were delivered by PROGMAR (Leszno, Poland). Hazelnut shells (H) obtained from the company AGRO Jarosław Sero-czyński (Nadbrzeż, Poland) were added at 2.5 wt.% to the appropriate, previously deducted amount of resin with curing agent. The hazelnut shells were ground using a MUKF-10 laboratory sieve mill (Młynpol P.P.H., Krzyżowa, Poland) equipped with a 0.2 mm mesh sieve. The resulting material was then dried in a Plus II incubator at 70 ± 2 °C for a minimum of 3 days [24,25], followed by the addition of 12.5 wt.% to the appropriate, previously deducted amount of resin with curing agent. Building on our previous research [26,27,28], the addition of 20 wt.% is the optimal amount to maintain high mechanical strength properties of the fabricated composites. A flame-retardant system consisting of 10 wt.% expanded graphite (EG) (Walter Thieme Handel GmbH, trading as Addforce FR EG) (Stade, Germany) and 10 wt.% ammonium polyphosphate (APP) (Walter Thieme Handel GmbH, trading as Addforce FR APP201) (Stade, Germany) was added to the previously deducted amount of resin with curing agent. Based on the manufacturer’s specifications, the APP consists of at least 31% phosphorus and 14% nitrogen (max. particle size of 15 µm), while the EG is characterized by a grain size of min. A total of 70% (50 mesh) undergoes a volume expansion of 250 mL/g.

The amount of fillers and the resin mixture was selected based on prior experimental trials. These amounts were considered optimal for thoroughly impregnating the fabrics while preventing void formation at the interfaces.

The epoxy resin components were mixed using a proLAB 075 mechanical stirrer (GlobimiX Ltd., Ząbkowice Śląskie, Poland) under subatmospheric pressure. Fabric layers measuring 330 × 330 mm^2^ were placed one by one on an ultra-high-molecular-weight polyethylene plate coated with Teflon film. For vacuum-bag processing, the epoxy resin was distributed uniformly using a roller. The lay-up was then covered with peel ply, a vacuum blanket, and a vacuum bag. Vacuum was applied to remove excess resin, and the samples were kept under vacuum until polymerization was complete. A similar amount of the epoxy resin and flame-retardant mixture (545 g) was used for each laminate. After forming, the laminates were cured at room temperature for 4 days and post-cured at 70 °C for 3 h. Finally, the composites were cut to the required dimensions for testing. All laminates had a thickness of 7.0 ± 0.5 mm.

The glass-fabric-reinforced composite (G) was selected as the reference formulation because glass fibers provide high tensile strength and stiffness and exhibit comparatively high thermal stability relative to the polymer matrix. Therefore, G provides a suitable baseline for evaluating the effects of hybrid reinforcement architectures on thermo-oxidative degradation and residue formation [29,30]. Each composite consisted of five two-layer fabric plies and was manufactured in one of the following configurations:

- carbon/aramid/glass/basalt/flax (CAGBF);
- aramid/carbon/glass/basalt/flax (ACGBF);
- glass (G).
- Some of the composite layers were replaced with a powdered filler:
- vermiculite + carbon/aramid/mikrobalon + glass/basalt/hazenut shells + flax (VCAMGBHF);
- vermiculite + aramid/carbon/mikrobalon + glass/basalt/hazelnut shells + flax (VACMGBHF);
- vermiculite + aramid/glass/carbon/basalt/hazelnut shells + flax (VAGCBHF).

The “+” symbol indicates that one layer was replaced with the corresponding filler. A description of the samples is provided in Table 1.

### 2.2. Methods

Cone calorimetry (CC) was performed using a cone calorimeter (Fire Testing Technology Ltd., East Grinstead, UK) in accordance with ISO 5660-1:2015 [31]. The samples (100 × 100 × 7 mm^3^) were wrapped in aluminum foil and exposed horizontally to a heat flux of 35 kW·m^−2^. Spark ignition was used to ignite the pyrolysis products. The residues were photographed using an EOS 400D digital camera (Canon Inc., Tokyo, Japan).

Smoke optical density was assessed using a smoke density chamber (Fire Testing Technology Ltd., East Grinstead, UK). The samples were exposed to a heat flux of 25 kW·m^−2^ without a pilot flame, in accordance with ISO 5659-2:2017 [32]. As in the cone calorimetry tests, the reported values are averages of three specimens from each series.

Fire-hazard testing was performed using the horizontal burning test (Method A) specified in IEC 60695-11-10:2013 [33], with classification based on the HB criteria. The classification was determined according to the standard’s criteria for linear burning rate and specimen thickness. The specimens (125 × 10 × 7 mm^3^) were exposed to a 50 W test flame. Burning behavior was assessed using the linear burning rate (v), calculated according to Equation (1):

(1) $$v=\left(\frac{L}{t}\right)\times (\frac{60\;s}{\min})$$

where L is the damaged length and t is the elapsed time for the flame to travel from the 25 mm mark past the 100 mm mark. 

A simultaneous thermal analyzer (STA 449F3 Jupiter, Netzsch, Selb, Germany) was used to investigate the thermal degradation of the prepared composites. Samples weighing approximately 10 mg were placed in alumina crucibles and heated from 30 to 900 °C at a rate of 10 °C·min^−1^. Thermal decomposition was studied under an air flow rate of 30 mL·min^−1^ over the sample, with nitrogen used as the protective gas at a flow rate of 20 mL·min^−1^. The gases and vapors released during STA measurements were transferred directly to a Fourier-transform infrared (FT-IR) spectrometer (Tensor 27, Bruker, Ettlingen, Germany) for STA/FT-IR analysis. The FT-IR instrument was coupled to the STA using a polytetrafluoroethylene transfer line maintained at 230 °C and a gas cell maintained at 230 °C to prevent condensation of the evolved gases. Spectra were recorded over the range of 4000–600 cm^−1^ at a resolution of 4 cm^−1^ as a function of temperature. Three samples from each series were analyzed.

Fire effluents generated during the combustion and thermal degradation of the selected materials were produced in a steady-state tube furnace (Purser furnace) using a procedure based on ISO/TS 19700:2016 [34], which describes a method for generating fire effluents under controlled decomposition conditions. Samples weighing 20 g each were placed in quartz boats and introduced into the heated zone of the furnace, which was maintained at 650 °C. The measurements were conducted at a primary air flow rate of 15 L·min^−1^ and a total air flow rate of 50 L·min^−1^. The experimental conditions were designed to simulate well-ventilated flaming combustion, characteristic of the initial stage of fire development.

The resulting gases were directed into a mixing chamber and withdrawn using a sampling probe. They were then transported through a heated transfer line maintained at 180 °C to a gas cell, also maintained at 180 °C, before being conveyed through a second heated transfer line to a Gasmet DX4000 gas analyzer (Gasmet Technologies Oy, Vantaa, Finland). The concentrations of selected gaseous compounds, including carbon dioxide (CO_2_), carbon monoxide (CO), hydrogen cyanide (HCN), nitrogen dioxide (NO_2_), nitric oxide (NO), ammonia (NH_3_), formaldehyde (HCHO), and hydrocarbons such as methane (CH_4_), ethane (C_2_H_6_), propane (C_3_H_8_), ethylene (C_2_H_4_), and hexane (C_6_H_14_), were measured using FT-IR spectroscopy. The analyzer was calibrated for the compounds listed above and connected to a computer for data acquisition and analysis. Three replicate tests were conducted for each material to assess the reproducibility of the emitted-gas measurements.

## 3. Results

### 3.1. Fire Behavior

**Figure 1:**
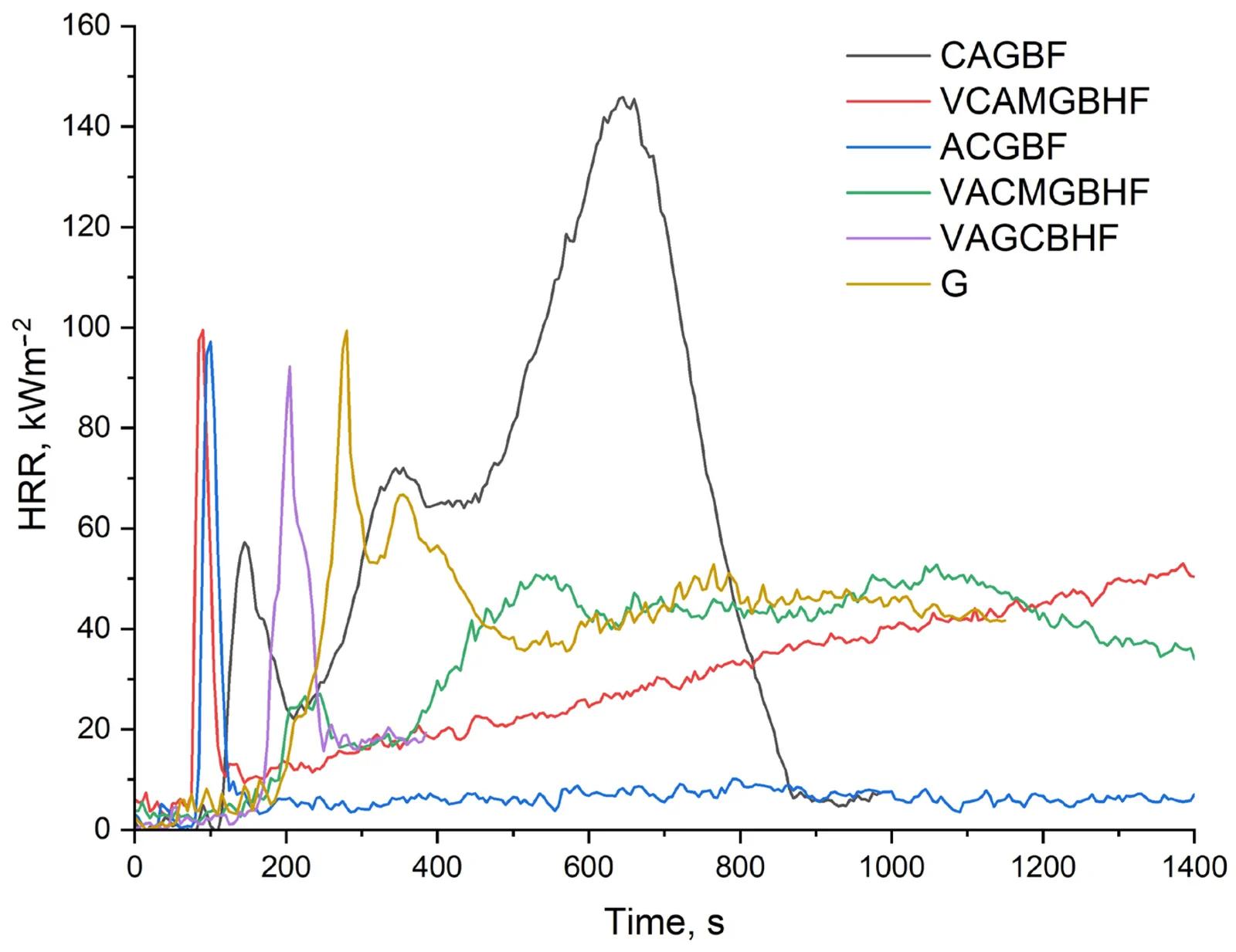
Representative curves of heat release rate of tested materials.

The combustion characteristics and reaction-to-fire properties of the hybrid composite systems were systematically evaluated using cone calorimetry based on the oxygen consumption principle. The heat release rate was determined from the oxygen consumed during combustion, following the relationship commonly referred to as Thornton’s rule [35]. The quantitative data extracted from the experimental profiles are summarized in Table 2, while the heat release rate (HRR) curves as a function of time are depicted in Figure 1. 

The time to ignition (TTI), representing the period required to initiate sustained flaming combustion on the specimen surface under a given heat flux [17], varied significantly among the formulations, ranging from 87 s for ACGBF to 206 s for VACMGBHF, compared to 139 s for reference sample G. The notable prolongation of TTI observed in VACMGBHF (206 s) and VCAMGBHF (186 s) demonstrates improved resistance to ignition during the early stages of thermal exposure. This delay can be attributed to the action of the intumescent flame-retardant system, which promotes the formation of a protective char layer and limits heat and mass transfer between the condensed and gas phases, thereby reducing the release of combustible volatile products and delaying the formation of an ignitable fuel–air mixture [36]. In contrast, the relatively short TTI of ACGBF indicates that this formulation was ignited rapidly under the applied test conditions, despite exhibiting favorable results for several subsequent combustion parameters.

The time to flameout (TTF) provides complementary information on the duration of flaming combustion under the specified test conditions. The reference sample G exhibited a TTF of 968 s, while considerably longer values were recorded for CAGBF (1038 s), VCAMGBHF (1320 s), VACMGBHF (1505 s), and VAGCBHF (1441 s). In contrast, ACGBF exhibited a markedly shorter TTF of 123 s. This result indicates that, although ACGBF ignited relatively quickly, sustained flaming was terminated much earlier than for the other tested formulations. The combination of a short TTF and low heat-release parameters suggests that the combustion of ACGBF was limited in duration and overall intensity. However, TTF should be interpreted together with the heat-release and mass-loss data, because flame extinction alone does not establish the extent of thermal decomposition or the amount of material consumed during the test [37,38].

The average heat release rate (av-HRR), representing a key parameter for characterizing the fire behavior and potential contribution of a material to fire growth [39], is calculated as the average HRR from the time of ignition to the end of the test. Av-HRR reached its highest value of 47 kW·m^−2^ for the reference sample G. The combination of the flame-retardant system and specific hybrid architectures led to a marked reduction in average HRR, decreasing to 32 kW·m^−2^ for VAGCBHF and reaching a minimum of 8 kW·m^−2^ for ACGBF. The exceptionally low av-HRR of ACGBF, approximately 83% lower than that of the reference sample, indicates a substantial reduction in the rate of heat release during combustion.

A concordant trend was observed for the peak heat release rate (pHRR), where the reference sample G registered 92 kW·m^−2^, whereas VACMGBHF exhibited a reduced pHRR of 79 kW·m^−2^. ACGBF, however, reached a pHRR of 97 kW·m^−2^, slightly exceeding the value measured for the reference sample. This result shows that the favorable behavior of ACGBF was not associated with a uniformly lower heat-release rate at every stage of the test. Instead, its fire response was characterized by a relatively high peak value combined with a very low average HRR (av-HRR) and a short time to peak heat release rate (t-pHRR) of 100 s. This combination suggests that heat release from ACGBF was concentrated within a comparatively short period rather than sustained over an extended combustion phase. By contrast, VACMGBHF showed the longest time to pHRR (1050 s) and a relatively low pHRR, consistent with a more gradual development of the heat-release process. Its ignition was also delayed (TTI = 206 s), and its time to flame-out was the longest among the tested materials (TTF = 1505 s). Together with the lower mass loss measured for VACMGBHF (16%), these results suggest that combustion and decomposition progressed over a longer period rather than producing an early, sharp heat-release peak. The multilayered residue observed after testing may have contributed to this behavior by restricting heat and mass transfer. The other modified hybrid composites also demonstrated prolonged heat-release development, with t-pHRR values of 783 s for VCAMGBHF and 258 s for VAGCBHF. For these formulations, the delayed heat-release peak is consistent with the protective action of the condensed-phase residue, which can limit heat and mass transfer and retard further thermal degradation of the underlying composite [40,41,42].

The maximum average rate of heat emission (MARHE), a critical indicator of potential fire spread rates [43], was 35 kW·m^−2^ for sample G. Notably lower MARHE values were obtained for ACGBF (20 kW·m^−2^) and VCAMGBHF (28 kW·m^−2^), reflecting a diminished capacity for fire propagation. Concurrently, the total heat release (THR), which is calculated as the average HRR from the time of ignition to the end of the test, decreased from 35 MJ·m^−2^ for the reference sample G to only 4 MJ·m^−2^ for ACGBF. This represents an approximately 89% reduction in total heat release relative to the reference material. The exceptionally low THR observed for ACGBF, together with its low average HRR and short TTF, indicates strongly restricted sustained combustion, even though its peak HRR was not lower than that of the reference sample. The results therefore suggest that the fire performance of ACGBF should be assessed in terms of the overall combustion process rather than on the basis of a single peak parameter.

The mass-loss results provide additional insight into this behavior. ACGBF exhibited a mass loss of 26%, compared with 10% for the reference sample G. The other formulations showed mass losses of 16% for VACMGBHF, 25% for VCAMGBHF, 25% for VAGCBHF, and 29% for CAGBF. Thus, the low THR and short TTF of ACGBF cannot be explained simply by a low mass loss. Rather, the comparison indicates that the amount of mass lost during the cone calorimeter test and the amount of heat released are not directly proportional across the tested formulations. Mass loss can result from several processes, including the release of volatile decomposition products and other gaseous species, as well as the breakdown of the polymer matrix and other composite constituents. In the case of ACGBF, the combination of a 26% mass loss and a THR of only 4 MJ·m^−2^ suggests that a considerable part of the mass-loss process did not translate into sustained, high-intensity heat release. This interpretation is consistent with the short TTF and low average HRR, although confirming the underlying mechanism would require additional evidence, such as characterization of the post-test residue and analysis of evolved gases.

The exceptionally low THR observed for ACGBF, together with its low average HRR and MARHE, may reflect the combined influence of the flame-retardant system and the specific reinforcement and filler architecture. One possible explanation is that the composition promotes the formation of a residue that restricts heat transfer or limits the transport of combustible degradation products to the flame. Another possibility is that the decomposition of the composite produces a substantial mass loss without generating an equivalent amount of heat through flaming combustion.

Smoke evolution characteristics were strongly influenced by the hybrid fabric architecture and particulate incorporation. Reference sample G yielded a total smoke release (TSR) of 219 m^2^·m^−2^ and a specific extinction area (SEA) of 316 m^2^·kg^−1^. ACGBF exhibited a substantially lower TSR of 123 m^2^·m^−2^, corresponding to a reduction of approximately 44% relative to the reference sample, while its SEA was 312 m^2^·kg^−1^, close to the reference value. This distinction is important because the lower TSR indicates a reduction in the total amount of smoke released during the test, whereas the similar SEA values suggest that the smoke production relative to the mass lost was not reduced to the same extent. Higher SEA values observed in certain flame-retardant formulations, such as 375 m^2^·kg^−1^ for VCAMGBHF, may be consistent with combustion conditions that favor the formation of smoke particles and unburned carbonaceous species [44].

As shown by the HRR profiles in Figure 1, the flame-retardant system and hybrid composite architectures influenced combustion behavior in different ways.

**Figure 2:**
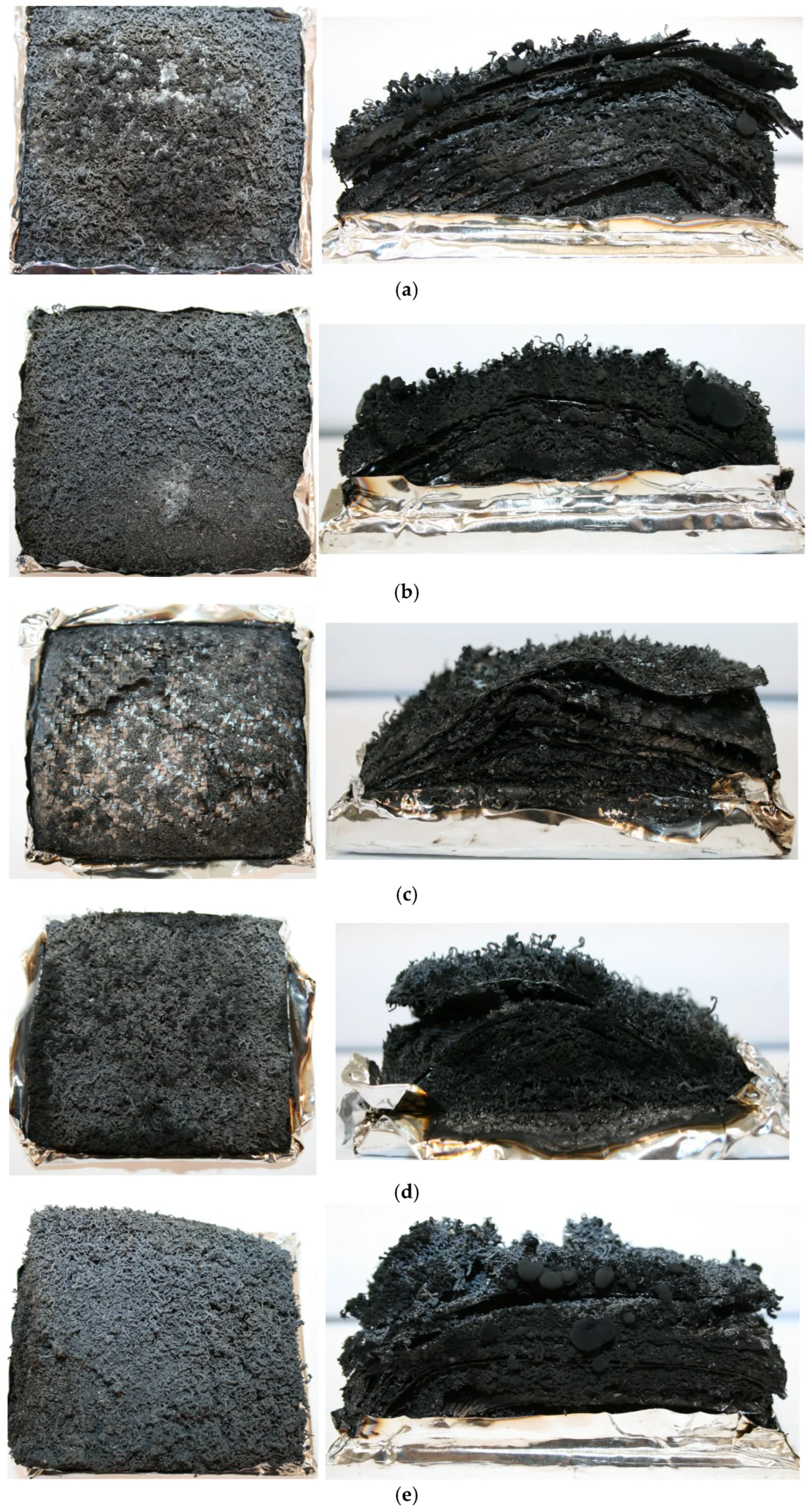

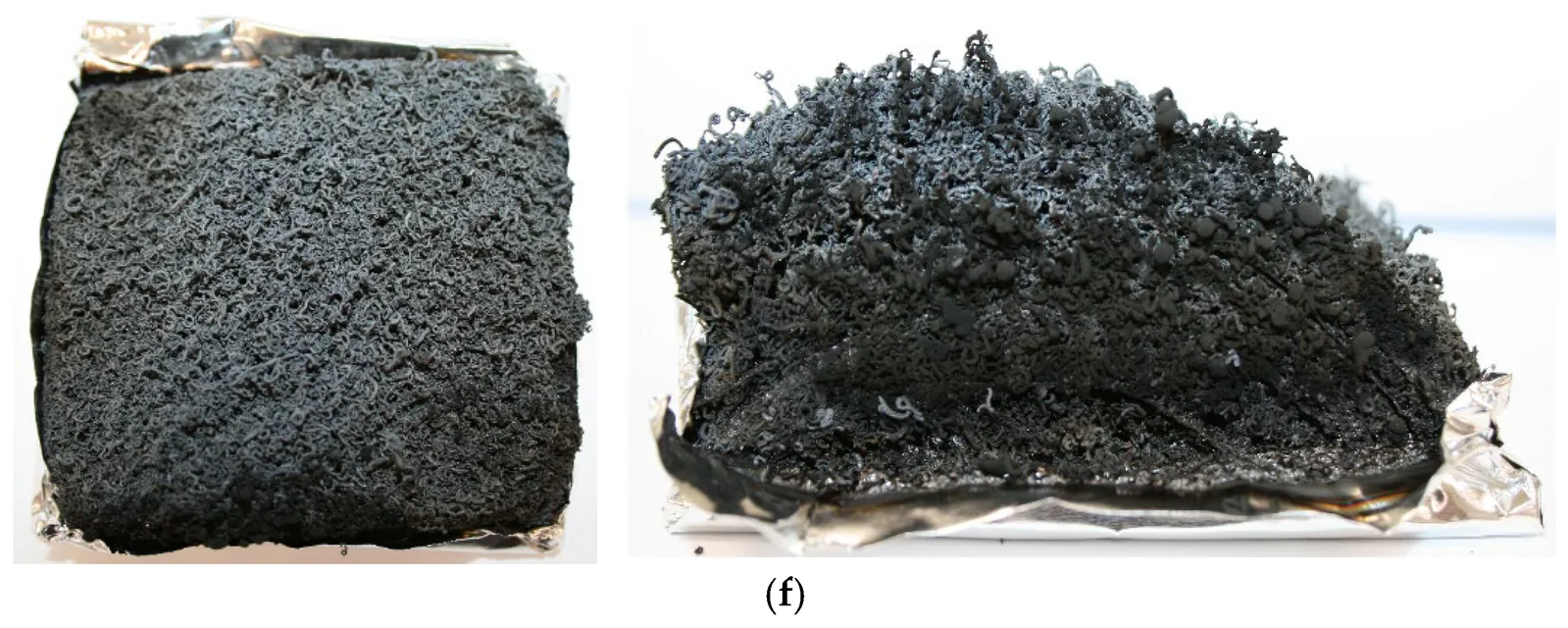
Photographs of residues after cone-calorimeter testing: (**a**) CAGBF, (**b**) VCAMGBHF, (**c**) ACGBF, (**d**) VACMGBHF, (**e**) VAGCBHF, (**f**) G. For each specimen, the left photograph shows the top view of the residue, whereas the right photograph shows its side view.

Visual evaluation of the post-test combustion residues (Figure 2a–f) provides direct visual evidence supporting the quantitative cone calorimetry results and illustrates the influence of the composite architecture on the formation and morphology of the protective residue. As shown in Figure 2, the morphology and extent of the residual protective layer varied considerably depending on the type and arrangement of the reinforcing fabrics and fillers. The char height of all tested composites ranged from 3 to 4 cm. Therefore, the analysis focused on the morphology of the resulting char layers.

According to our previous studies on related materials [27,28], carbon (C), oxygen (O), and nitrogen (N) are commonly detected in the residues formed during the thermal degradation of these types of materials, while phosphorus (P) may also remain when phosphorus-containing compounds are present in the formulation. The relative abundance and distribution of these elements may influence the chemical composition and structure of the resulting residue and, consequently, its ability to act as a protective barrier. In the present study, however, the elemental composition of the individual residues was not directly determined. Therefore, any relationship between elemental composition, char morphology, and cone calorimetry results should be considered a possible explanation rather than a confirmed causal mechanism.

The reference composite G (Figure 2f), consisting exclusively of glass fabric reinforcement, retained a predominantly inorganic glass-fabric network after exposure to the cone calorimeter. Although the flame-retardant system was present in the resin, the absence of additional hybrid fillers and reinforcing fabrics resulted in a different residue morphology, with less pronounced expansion and a less evident cohesive carbonaceous–mineral char structure than those observed in some of the hybrid composites. This observation is consistent with the calorimetric results, in which G exhibited an av-HRR of 47 kW·m^−2^, pHRR of 92 kW·m^−2^ and THR of 35 MJ·m^−2^. 

In contrast, the hybrid flame-retardant formulations (VCAMGBHF, ACGBF, VACMGBHF, and VAGCBHF; Figure 2b–e) developed more pronounced, voluminous, and structurally cohesive carbonaceous–mineral residues. In particular, ACGBF (Figure 2c) formed a relatively compact and uniform protective layer on the exposed surface, consistent with its exceptionally low THR of 4 MJ·m^−2^ and av-HRR of 8 kW·m^−2^. The cross-sectional views of VCAMGBHF (Figure 2b), VACMGBHF (Figure 2d), and VAGCBHF (Figure 2e) revealed expanded, multilayered residue structures, with char deposits retained between the reinforcing fabric plies. Such continuous and cohesive char structures may form an effective condensed-phase barrier that limits heat and mass transfer, reduces heat feedback to the underlying material, and restricts the release and transport of combustible volatile pyrolysis products [39].

The differences in calorimetric performance may therefore reflect not only the visible morphology of the residues but also their chemical composition and structural integrity. In particular, differences in the relative amounts of carbonaceous material and inorganic constituents, potentially including phosphorus-containing species, could affect the continuity, stability, and barrier efficiency of the protective layer. A more effective barrier may contribute to lower heat release by restricting the transfer of heat and combustible decomposition products. Overall, the observations indicate that, despite the use of the same general flame-retardant system, the architecture and composition of the hybrid reinforcement substantially influenced the formation and apparent protective effectiveness of the residue layer.

Smoke generation during combustion was evaluated using a smoke density chamber. The corresponding optical-density parameters, such as maximum specific optical density (Ds), cumulative optical density during the first 4 min (VOF4), mass loss, and horizontal-burning classification, are summarized in Table 3.

The specific optical density (Ds) measures the light attenuation caused by accumulated smoke effluent in a sealed chamber, whereas VOF4 quantifies the smoke accumulation rate during the early stage of combustion [32].

As shown in Table 3, smoke generation varied considerably among the investigated composite architectures. The reference composite G, consisting exclusively of glass fabric reinforcement, exhibited a maximum specific optical density (Ds) of 42. Compared with the reference composite G, higher Ds values were recorded for CAGBF (142), VCAMGBHF (67), ACGBF (82), and VACMGBHF (57), whereas the lowest value was observed for VAGCBHF (51). Thus, the maximum smoke optical density of CAGBF was approximately 3.4 times higher than that of the reference composite G. The differences in smoke generation cannot be attributed to the presence or absence of the flame-retardant system, since all investigated composites contained the EG/APP system. Instead, they indicate a pronounced influence of the reinforcing fabric configuration and the incorporation of additional fillers on smoke formation and retention.

The early-stage smoke accumulation parameter VOF4 also differed among the formulations. The reference composite G exhibited a VOF4 value of 34, while the corresponding values for the hybrid formulations ranged from 13 for ACGBF to 54 for VCAMGBHF. These differences demonstrate that the composite architecture affected not only the maximum smoke optical density but also the temporal characteristics of smoke generation. In particular, the lower Ds value of G compared with most hybrid formulations may be associated with its glass-fabric-only reinforcement architecture, whereas the incorporation of organic and mineral fillers and additional fabric types altered the thermal decomposition and smoke-forming processes.

**Figure 3:**
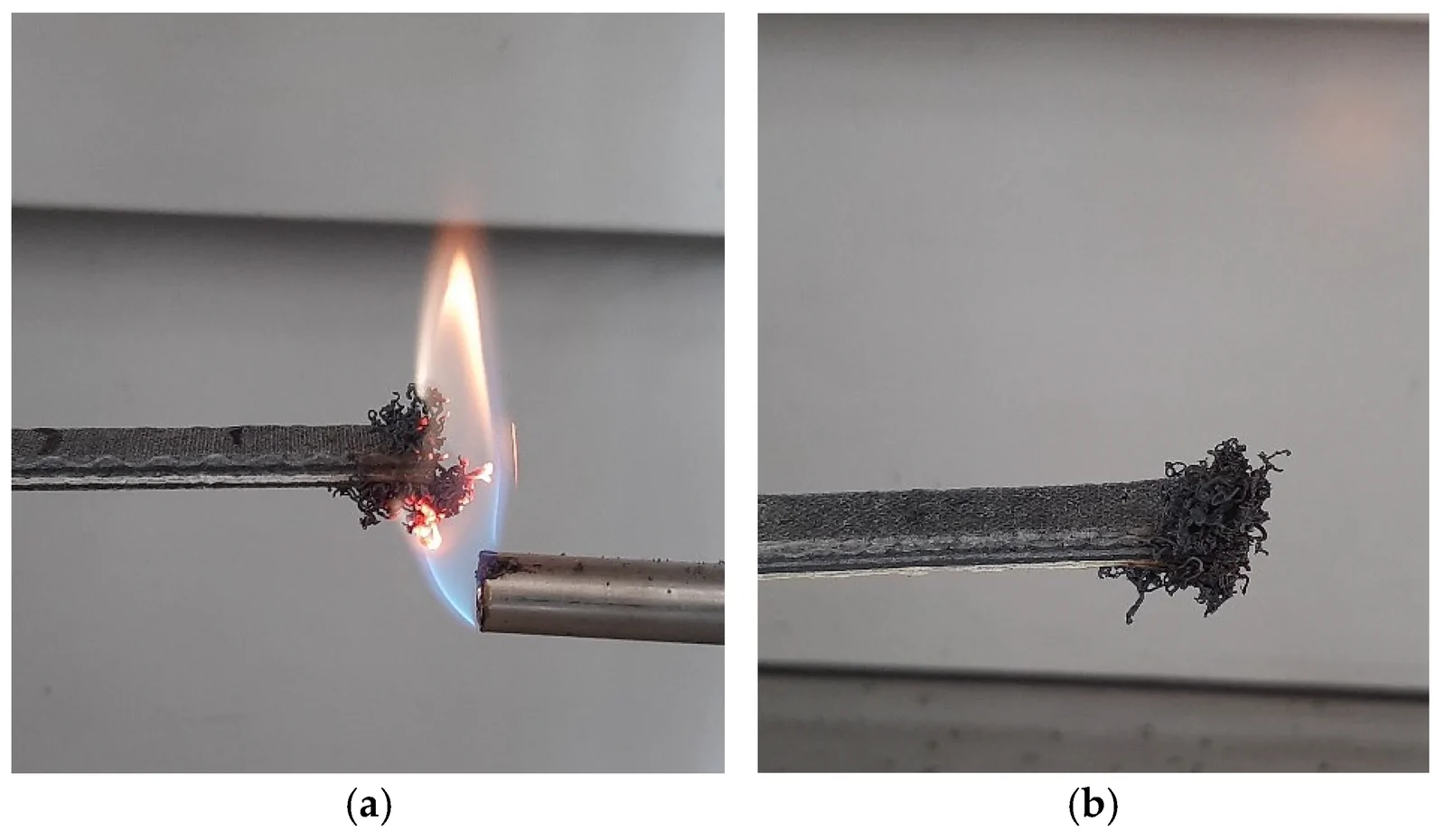
Photographs of flame-retardant samples during the horizontal burning test: (**a**) CAGBF and (**b**) ACGBF.

The flame-propagation behavior and fire hazard of the composite materials were evaluated using horizontal burning tests (Table 3 and Figure 3).

As summarized in Table 3, all tested composite formulations, including the glass-fabric reference composite G and the hybrid formulations CAGBF, VCAMGBHF, ACGBF, VACMGBHF, and VAGCBHF, met the criteria for the HB (horizontal burning) classification. Notably, the flame extinguished immediately after the burner was switched off in all tested specimens. This behavior may be associated, at least in part, with the presence of non-combustible inorganic fabric reinforcements, which can provide a thermally stable supporting network within the composite and help limit flame propagation [45]. Previous studies on fiber-reinforced polymer composites have shown that inorganic glass and basalt fibers can improve resistance to flame spread and help maintain composite integrity during thermal exposure [46].

As illustrated in Figure 3, the flame remained localized and did not propagate along the modified composite strip. This behavior may reflect the combined effects of the inorganic fiber network and the formation of a protective carbonaceous char layer at the exposed edge. Such a condensed-phase barrier may reduce heat transfer to the underlying polymer matrix and restrict the release and transport of combustible volatile pyrolysis products into the flame zone [47].

Although all tested formulations contained the same general flame-retardant system, they differed in the extent and morphology of the post-combustion residues. These differences may be associated with the type, arrangement, and proportion of the reinforcing fabrics and fillers, which can influence heat transfer, volatile release, and the formation of protective condensed-phase residues.

### 3.2. Thermal Decomposition

**Figure 4:**
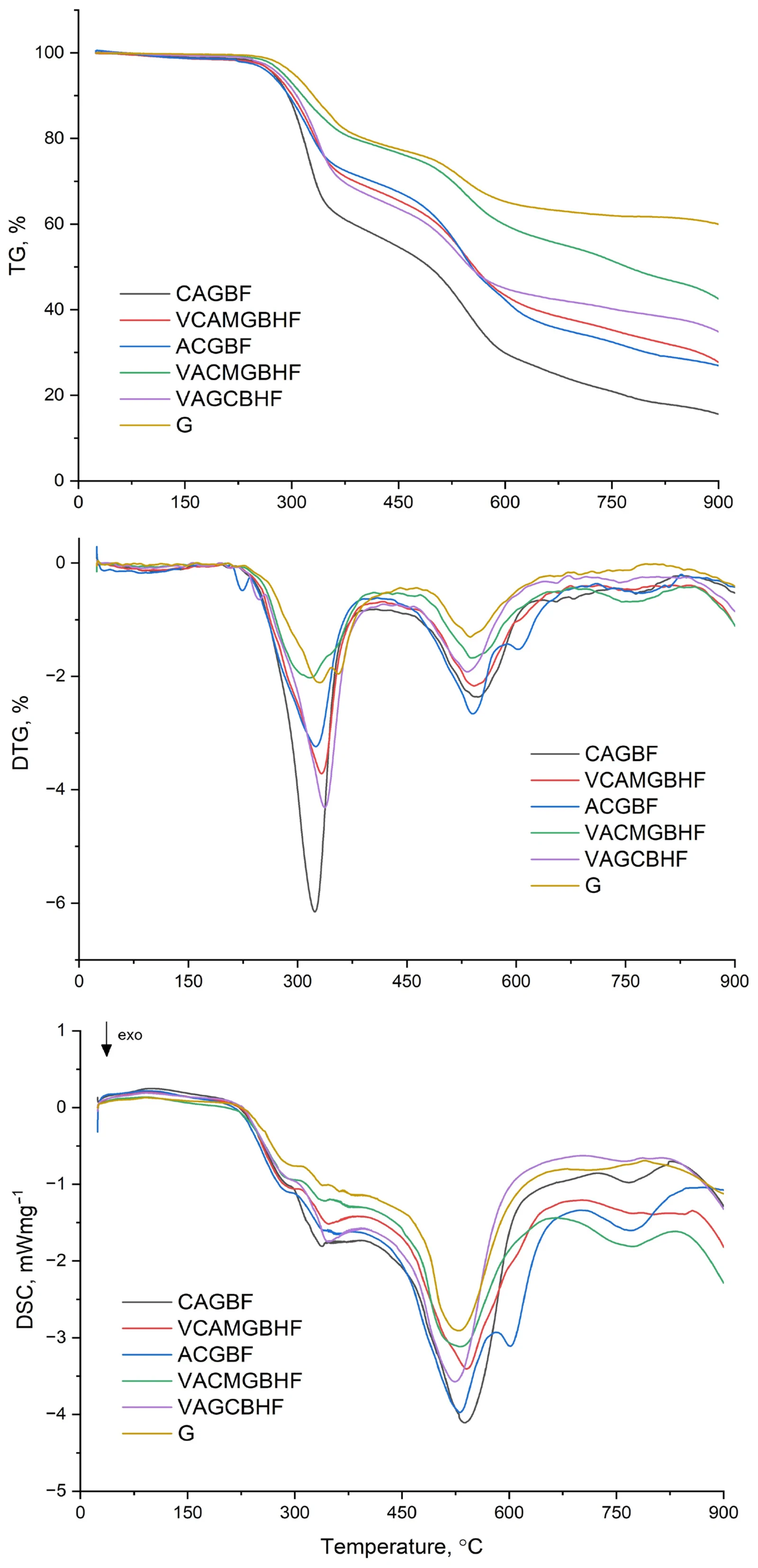
TG, DTG and DSC curves of tested composites.

Thermal properties of the tested materials were analyzed using thermogravimetry (TG), derivative thermogravimetry (DTG), differential scanning calorimetry (DSC), and three-dimensional FT-IR analysis of evolved gases (STA/FT-IR). The results demonstrated that the composite structure significantly affected thermal stability and thermo-oxidative degradation behavior. As summarized in Table 4 and shown in the TG, DTG, and DSC curves (Figure 4), the samples exhibited composition-dependent, multistage thermal decomposition.

In Table 4, the characteristic thermo-oxidative degradation temperatures (T1–T7), corresponding to the DTG peak temperatures, the associated mass losses (m1–m7), the maximum mass-loss rates, and the residual masses at 900 °C (U_900_) are summarized. The reference composite G exhibited a multistage thermo-oxidative degradation process, with the first distinct degradation peaks occurring at approximately 331 °C. It displayed two early mass-loss steps (m2 = 13.0% at 331 °C and m3 = 9.4% at 356 °C), which may reflect overlapping contributions from the thermal degradation of the epoxy (EP) matrix, APP-related acid-catalyzed reactions, and the expansion and thermal decomposition of EG [48,49,50,51,52]. Upon heating, APP releases gaseous products, including ammonia and water, and forms condensed phosphorus-containing species that can promote dehydration and char-forming reactions in the polymer matrix [48,50]. EG can also undergo expansion and influence the thermal degradation behavior and residue formation in epoxy composites [53,54,55]. The subsequent major mass-loss stage occurred at T4 = 535 °C (m4 = 18.8%), with a maximum thermo-oxidative degradation rate of 1.30%·min^−1^. Notably, composite G retained the highest residual mass at 900 °C (U_900_ = 58.8%). This high residual mass can be primarily associated with the relatively high fraction of thermally stable glass-fiber reinforcement, although contributions from carbonaceous and phosphorus-containing residues generated by the EP/APP/EG system should also be considered [56].

The hybridization of the fiber reinforcement in CAGBF, VCAMGBHF, ACGBF, VACMGBHF, and VAGCBHF markedly altered the thermo-oxidative degradation pathways and residue formation behavior. A distinct difference among the formulations was observed in the lower-temperature regime (T1 ≈ 234–251 °C), where ACGBF and VAGCBHF exhibited an early and relatively small mass loss (m1 = 2.5% and 2.0%, respectively). This initial stage may be associated with the early thermal decomposition of APP, which can involve the release of NH_3_ and H_2_O and the formation of condensed phosphorus-containing species [57]. In contrast, CAGBF, VCAMGBHF, and VACMGBHF did not exhibit a distinct T1 event, suggesting that any initial mass loss was either too small to resolve or overlapped with subsequent degradation events under the applied test conditions. This difference may result from variations in fiber architecture, matrix/additive distribution, and local heat-transfer conditions within the hybrid laminates.

All hybrid composites underwent a major thermo-oxidative degradation stage between approximately 320 °C and 343 °C, with mass losses ranging from 21.4% to 42.0%. Significant differences in the maximum thermo-oxidative degradation rates were observed among the formulations. CAGBF exhibited the most pronounced mass-loss event, with a mass loss of 42.0% and a maximum decomposition rate of 6.21%·min^−1^ at 332 °C. In contrast, VACMGBHF showed a substantially lower maximum decomposition rate of 2.03%·min^−1^ at 320 °C, together with a lower mass loss of 21.4%. This behavior indicates a less pronounced mass-loss event in VACMGBHF and may reflect the combined effects of its specific fiber architecture and the EG/APP flame-retardant system on the heat and mass transfer processes occurring during degradation [48].

Major differences were also observed at elevated temperatures above 600 °C. Whereas CAGBF, VCAMGBHF, and VAGCBHF completed their main thermo-oxidative degradation processes by approximately 550 °C, ACGBF and VACMGBHF exhibited additional high-temperature mass-loss stages. ACGBF showed additional degradation steps at T5 = 612 °C (m5 = 6.6%) and T6 = 780 °C (m6 = 10.9%), whereas VACMGBHF exhibited degradation extending to T6 = 751 °C (m6 = 8.5%) and T7 = 925 °C (m7 = 8.1%). These high-temperature mass-loss events may be associated with the further oxidation of carbonaceous residues derived from the EP matrix and EG, as well as transformations of the remaining phosphorus-containing and inorganic phases [51,53,54,55]. However, TGA/DTG results alone do not allow these individual events to be assigned unambiguously to specific constituents. Therefore, the extended thermo-oxidative degradation range observed for ACGBF and VACMGBHF should be interpreted as evidence of additional high-temperature mass-loss processes rather than direct proof of a specific anchoring mechanism between the fiber network and the intumescent phase.

Consequently, the final residual masses at 900 °C (U_900_) reflect the combined contributions of the inorganic fiber fraction and the thermally stable residues that remain after thermo-oxidative degradation. Among the hybrid formulations, VACMGBHF achieved the highest residual mass (U_900_ = 38.8%), followed by VAGCBHF (32.6%) and ACGBF (26.2%), whereas CAGBF exhibited the lowest residual mass (U_900_ = 14.4%). These differences reflect not only the extent of primary matrix degradation but also the composition and thermal stability of the inorganic and carbonaceous fractions remaining under the applied test conditions [53,54]. Overall, the results indicate that, while the EG/APP system provides the principal chemical basis for intumescent char formation and flame-retardant action, the fiber composition and hybrid architecture can substantially influence the thermo-oxidative degradation kinetics and the stability of the resulting protective residue [50].

The DSC curves (Figure 4) reveal prominent exothermic effects associated with the thermo-oxidative degradation of the composites and the oxidation and transformation of the thermally generated residues. All composites exhibited predominantly exothermic behavior over the investigated temperature range up to 900 °C. These effects are broadly consistent with the thermal degradation stages identified by TG/DTG analysis. Similar relationships between thermal degradation stages and DSC/DTA signals have been reported for flame-retardant epoxy and fiber-reinforced polymer composites [58,59]. Nevertheless, individual DSC effects should be interpreted together with the TG/DTG results because overlapping polymer degradation, oxidation, volatilization, and transformations of residual phases may contribute to the measured heat-flow signal [58,60].

In the initial temperature region up to approximately 220 °C, minor baseline shifts and weak thermal effects are observed. These features may be associated with moisture desorption, the release of low-molecular-weight compounds, and initial thermal conditioning of the samples. Between 300 °C and 380 °C, a distinct exothermic region appears in all samples. Under the applied oxidative conditions, this response may reflect the overlapping effects of polymer-matrix degradation, oxidation reactions, and transformations involving the flame-retardant components. Ammonium polyphosphate (APP) undergoes thermal decomposition, releasing ammonia and water and generating phosphorus-containing acidic species that can promote condensed-phase char formation [48,49,58]. In the presence of air, these processes may overlap with oxidation of the decomposing polymer and the developing carbonaceous residue. Sample CAGBF exhibits the most pronounced exothermic response in this temperature range, around 335–350 °C, with a reported signal magnitude of approximately −1.8 mW·mg^−1^. This thermal effect coincides with substantial mass loss in the TG data (m2 = 42.0%), suggesting that intensive degradation and associated chemical reactions occur within this interval. In contrast, samples VACMGBHF and the reference composite G exhibit weaker exothermic effects, indicating a lower magnitude of the net heat-release signal under the measurement conditions.

The principal thermal event for all composite formulations appears as a sharp, pronounced exothermic peak between 500 °C and 560 °C, with peak maxima at approximately 525–540 °C. Given the oxidative measurement atmosphere, this event may be associated with oxidation of the remaining organic material and carbonaceous residue, accompanied by further thermal degradation and transformations of the inorganic fraction. In composites containing expandable graphite, the observed thermal response may also involve transformations of the graphite-containing residue and reactions between the residual phases and oxygen [59]. CAGBF and ACGBF exhibit the largest reported peak values, approximately −4.1 and −4.0 mW·mg^−1^, respectively. Intermediate values are observed for VAGCBHF (−3.6 mW·mg^−1^) and VCAMGBHF (−3.4 mW·mg^−1^), whereas VACMGBHF (−3.1 mW·mg^−1^) and the reference composite G (−2.9 mW·mg^−1^) exhibit smaller peak magnitudes. These differences indicate variations in the intensity of the net thermal response during the principal high-temperature degradation stage. The smaller peak magnitude observed for VACMGBHF may reflect less intense heat-releasing reactions within this temperature interval. However, it does not independently demonstrate a lower degradation rate or a more effective protective-barrier mechanism [58,59].

Above 600 °C, the DSC curves reveal additional thermal effects that distinguish the high-temperature behavior of the hybrid composites. Samples CAGBF, VAGCBHF, and G exhibit broad, relatively stable thermal-response regions between 650 °C and 850 °C, whereas ACGBF displays a secondary sharp exothermic peak at approximately 770–780 °C, with a reported value of about −1.6 mW·mg^−1^. VACMGBHF exhibits a sustained thermal response extending to 900 °C, with a secondary feature around 760–770 °C. Under the applied airflow conditions, these high-temperature effects may be associated with further oxidation of residual carbonaceous material and thermal transformations of the remaining inorganic phases. Their occurrence should be considered alongside the late-stage mass-loss steps identified in the TG analysis, particularly T6 and T7 for ACGBF and VACMGBHF. Similar high-temperature thermal effects have been discussed in studies of flame-retardant epoxy composites and phosphorus-containing systems, although the precise mechanisms depend on residue composition and experimental conditions [59]. Accordingly, the DSC signals above 600 °C indicate continued thermal activity within the residual phase but do not, by themselves, establish a specific structural-reorganization mechanism. The extended thermal response of VACMGBHF, together with its relatively high residual mass at elevated temperatures, suggests that a substantial fraction of thermally stable material remains after the main degradation stages. Nevertheless, residual mass and DSC response provide complementary rather than interchangeable information. TG quantifies mass retention, whereas DSC records the net heat-flow effects associated with processes occurring during heating [45,56].

**Figure 5:**
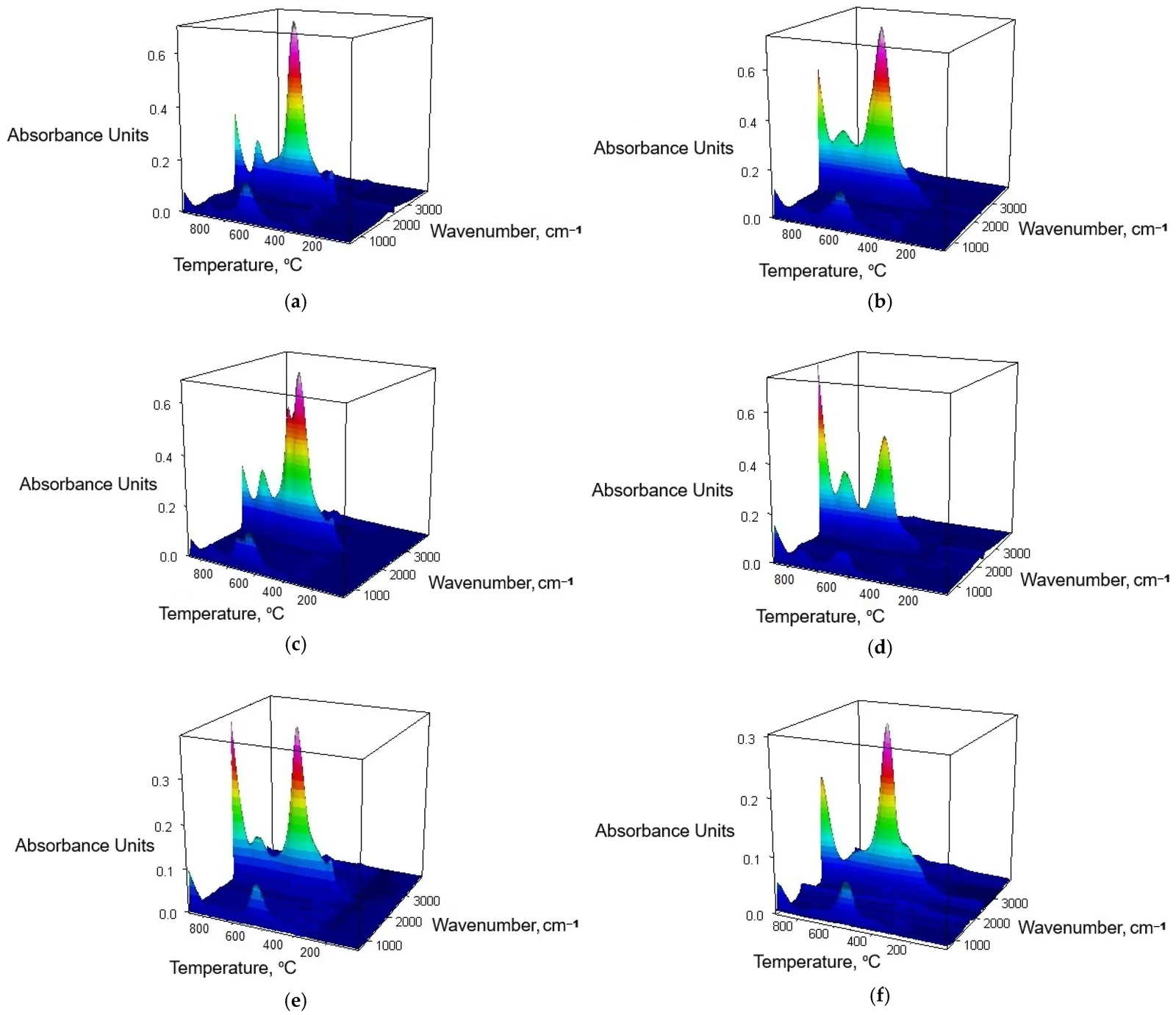
3D infrared spectrum of the emitted gases for (**a**) CAGBF, (**b**) VCAMGBHF, (**c**) ACGBF, (**d**) VACMGBHF, (**e**) VAGCBHF, (**f**) G.

STA/FT-IR analysis provided insight into the thermal decomposition of the hybrid composites. The measurements were conducted with an air flow rate of 30 mL·min^−1^ over the sample. The three-dimensional FT-IR spectra recorded throughout the thermal decomposition process are presented in Figure 5.

The interpretation of the 3D FT-IR spectra of evolved gases (Figure 5) further clarifies the decomposition mechanism operating in both the gas and condensed phases. Across all composite formulations, the overall profile of gaseous emissions closely follows the characteristic mass-loss steps recorded in the TG analysis. Such correspondence between the TG mass-loss stages and the evolution of gaseous products is characteristic of TG-FTIR analysis and has been reported for epoxy-based flame-retardant composites [61].

In the lower temperature regime (300–380 °C), an initial emission region is observed, primarily characterized by absorption bands corresponding to ammonia (NH_3_, 930–965 cm^−1^) and water vapor (H_2_O, 3500–4000 cm^−1^). The evolution of NH_3_ in this temperature range is consistent with the thermal decomposition of APP, whereas the broad absorption region around 3500–4000 cm^−1^ can be associated with water vapor and hydroxyl-containing volatile products [52]. Similar TG-FTIR investigations of APP-containing polymer systems have identified NH_3_ as a characteristic gaseous product of the initial APP decomposition stage [62]. The formation of phosphorus-containing condensed-phase species during APP decomposition may additionally promote char formation and modify the subsequent evolution of volatile degradation products [52].

The main degradation phase (500–560 °C) is dominated by intense absorption peaks around 2300–2380 cm^−1^, characteristic of carbon dioxide (CO_2_), and bands in the region of approximately 2100–2200 cm^−1^, associated with carbon monoxide (CO). These signals are accompanied by C–H stretching vibrations in the range of 2850–3000 cm^−1^, indicating the evolution of hydrocarbon-containing volatile products during degradation of the polymer matrix [61]. Recent TG-FTIR studies of epoxy-based flame-retardant systems have demonstrated that the incorporation of APP-based flame retardants can substantially reduce the intensity of gaseous hydrocarbon, aromatic, CO and CO_2_ signals compared with the unmodified epoxy matrix, indicating a reduction in the evolution of volatile degradation products [52]. Similar behavior has been reported for APP-containing polymer systems, in which the gas-phase products evolved during the main decomposition stage were strongly influenced by the formation of a carbonaceous condensed-phase residue [49].

A clear difference in gaseous emission intensity is evident among the investigated samples. CAGBF (a) and ACGBF (c) exhibit the highest overall absorbance amplitudes (exceeding 0.6 a.u.), indicating a greater intensity of evolved-gas release during the corresponding degradation stages. In contrast, composite VACMGBHF (d) displays substantially lower absorbance intensities across the main release stages, with peak values remaining below 0.4 a.u. A comparable reduction in the intensity of gaseous products has been observed in recent 3D TG-FTIR studies of flame-retardant epoxy composites, where lower absorption intensities were associated with reduced release of combustible organic volatiles [61]. In the present study, the lower gaseous emission intensity observed for VACMGBHF is consistent with its lower decomposition rate during the main TG degradation stage and its relatively high residual mass at elevated temperatures. This behavior may be related to the combined influence of the hybrid fiber architecture and the APP/EG flame-retardant system, which can promote the formation and stabilization of a condensed-phase residue and thereby restrict the evolution and transport of volatile degradation products [48]. However, the FT-IR results alone do not directly demonstrate the formation or morphology of a dense protective char barrier; rather, they provide complementary evidence of reduced volatile release that is consistent with the condensed-phase protection mechanism identified from the TG/DTG, DSC and cone-calorimetry results.

### 3.3. Analysis of Evolved Combustion Products

The release of gaseous thermal-decomposition products, including asphyxiant and irritant gases and hydrocarbons, was investigated under standardized steady-state conditions [34] at 650 °C and an air flow rate of 15 L·min^−1^. These conditions simulate well-ventilated flaming combustion.

The released substances were analyzed in hybrid composites with different fabric and filler compositions. The aim was to identify differences in the gaseous products generated during combustion of the various composite formulations.

**Figure 6:**
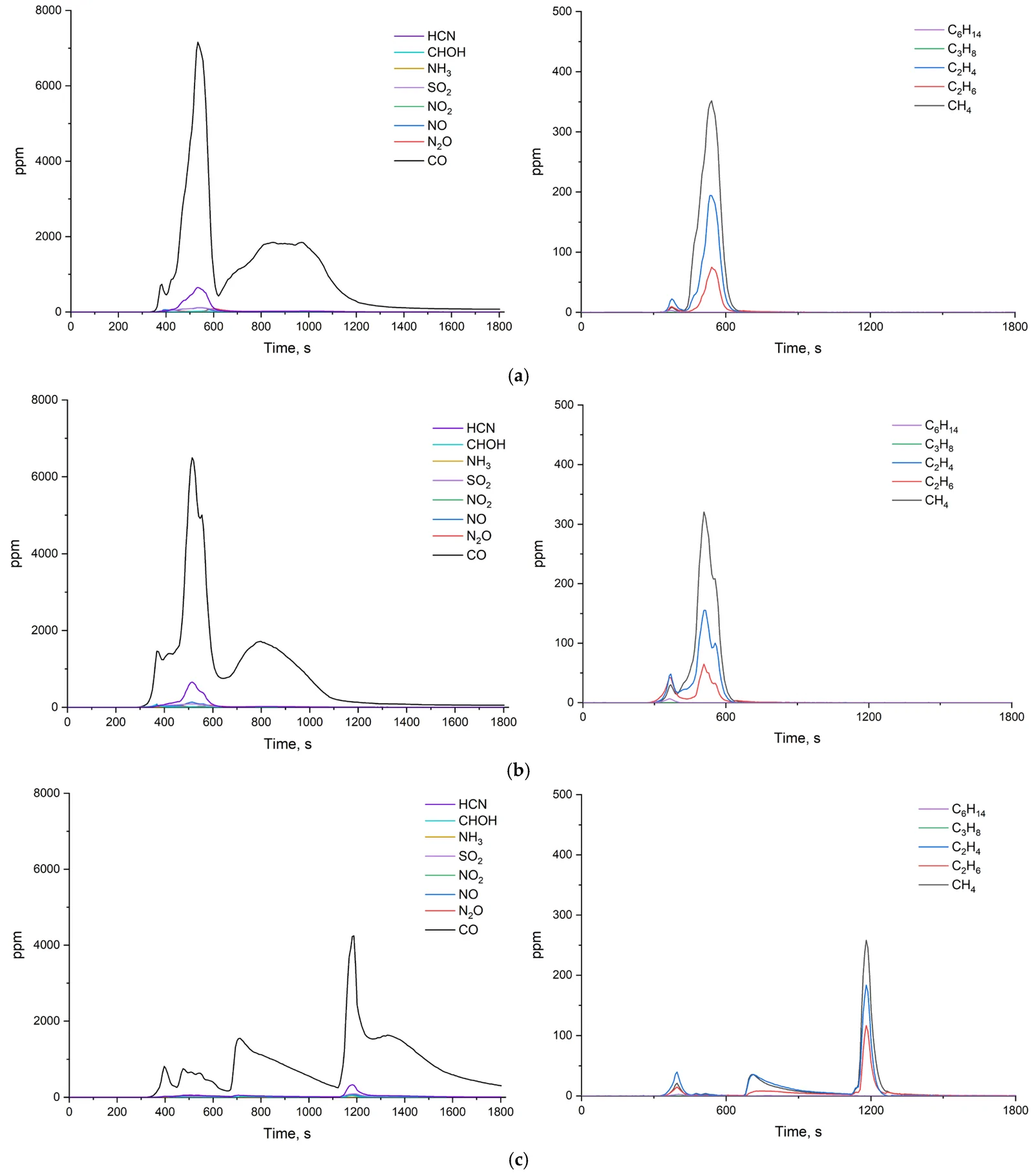

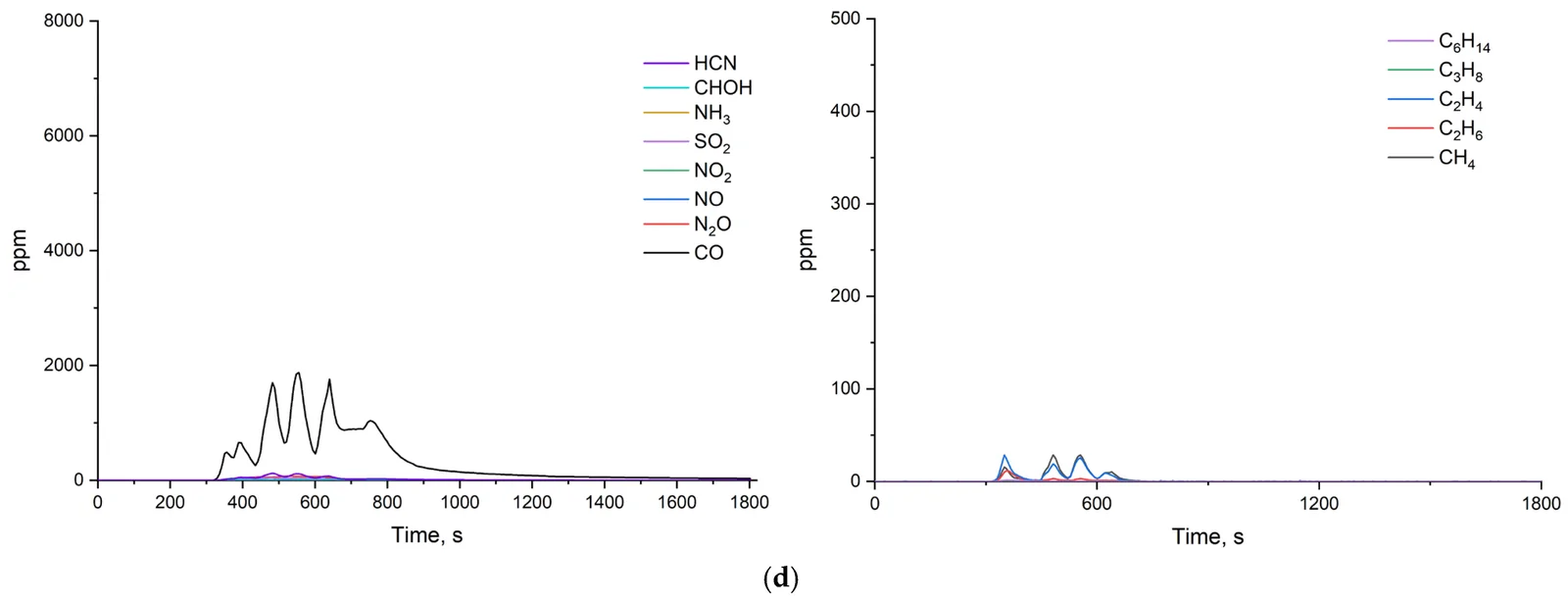
Representative emission profiles of asphyxiant and irritant gases and light hydrocarbons generated by the hybrid composites during steady-state tube-furnace tests under well-ventilated flaming conditions: (**a**) VACMGBHF, (**b**) VAGCBHF, (**c**) ACGBF, and (**d**) G.

The concentrations of asphyxiant and irritant gases released during the thermal decomposition and combustion of the hybrid composite samples are presented as a function of time in Figure 6.

An analysis of the recorded time curves in Figure 6 indicates significant differences in the composition and kinetics of gaseous product emissions between the glass-fabric reference composite (G) and the hybrid composites (VACMGBHF, VAGCBHF, and ACGBF). Such differences are consistent with the strong dependence of gas-phase fire behavior on the composition of the polymer matrix, the type and content of inorganic reinforcement, and the nature of the flame-retardant system [59,60,63].

Composite G exhibited comparatively lower concentrations of selected asphyxiant and irritant gases and light hydrocarbons than the investigated hybrid systems. However, this comparison should be interpreted with caution because the composites differed in their reinforcement and filler compositions and, consequently, in the relative proportions of combustible and non-combustible constituents. The relatively low intensity of gaseous emissions observed for G may be associated with its high glass-fiber content and the presence of the flame-retardant system. Recent studies on epoxy/glass-fiber composites have demonstrated that glass fibers substantially modify the decomposition and combustion behavior of the epoxy matrix by replacing part of the combustible fraction and altering the amount and nature of the condensed-phase residue [59]. In particular, glass fibers may reduce the amount of polymer available as fuel and modify carbonaceous-char formation, thereby affecting both heat-release characteristics and the evolution of gaseous degradation products [60]. Therefore, the comparatively lower gas-emission intensity observed for G should be interpreted in terms of the combined effects of matrix dilution, inorganic reinforcement, and flame-retardant action rather than as evidence of intrinsically “cleaner” combustion.

The introduction of the hybrid systems VACMGBHF, VAGCBHF, and ACGBF modifies the thermo-oxidative degradation and combustion behavior of the composites and is accompanied by increased emissions of light hydrocarbons and selected incomplete-combustion products. The formation of a more complex carbonaceous–mineral residue in the hybrid systems may alter heat and mass transfer within the degrading composite and influence the accessibility of gaseous pyrolysis products to the reaction zone [47]. Recent studies of flame-retardant epoxy systems have shown that condensed-phase protection can reduce the release of combustible organic volatiles, while the composition and morphology of the resulting residue strongly influence the subsequent gas-phase combustion behavior [47]. Accordingly, the higher concentrations observed for selected gaseous products in the hybrid composites may reflect changes in the balance between condensed-phase retention of degradation products and their subsequent oxidation in the flame zone. These differences should also be considered in relation to the different proportions of combustible polymer matrix, organic fillers and non-combustible reinforcement components in the individual formulations. 

The flame-retardant components may additionally contribute to gas-phase flame inhibition through the release of non-combustible or weakly combustible gaseous species and through interactions with the radical reactions responsible for flame propagation [64]. Nitrogen-containing flame-retardant components can release species such as NH_3_ and nitrogen-containing gases, which may dilute combustible volatiles and modify the local gas-phase composition and temperature. Phosphorus-containing flame retardants can also influence flame chemistry through gas-phase radical interactions, while simultaneously promoting condensed-phase char formation [64]. These effects can alter the oxidation efficiency of volatile intermediates and may contribute to the observed changes in the temporal profiles of CO, light hydrocarbons and other gaseous products.

The increased emission of nitrogen-containing gases observed for selected hybrid composites is consistent with the presence of additional nitrogen-containing components and functional modifiers in their formulations. During thermo-oxidative degradation and combustion, nitrogen-containing structures can generate species such as NH_3_, HCN and NO_x_, with their relative yields depending strongly on the chemical structure of the polymer, nitrogen-containing additives, temperature and oxygen availability [65]. Recent experimental studies on epoxy-based materials confirm that HCN, CO and nitrogen oxides may occur simultaneously in fire effluents and that their concentrations are strongly influenced by the formulation and combustion conditions [65]. Therefore, the higher nitrogen-containing gas emissions observed for the hybrid composites can reasonably be associated with their formulation and the altered combustion environment. However, the present measurements do not allow the formation pathway of HCN to be assigned unequivocally to a specific component, nor do they provide sufficient evidence to directly confirm a fuel-NO_x_ mechanism. Such mechanistic assignments would require additional analysis of the nitrogen balance and/or measurements performed under controlled oxygen concentrations.

The presence of sulfur dioxide (SO_2_) in the flue gases generated during combustion of the investigated hybrid composites can be associated primarily with the sulfur-containing intercalation chemistry of the expanded graphite (EG) incorporated into their formulations. Expanded graphite is commonly produced by intercalation of natural graphite with sulfuric acid or other sulfur-containing intercalants, followed by thermal expansion. During this process, although a substantial fraction of the intercalant is removed during processing and expansion, residual sulfur-containing species may remain within the graphite structure. Recent thermal-volatilization studies of graphite intercalation compounds containing sulfuric acid have demonstrated that SO_2_ is released during subsequent heating, with pronounced SO_2_ evolution at elevated temperatures attributed to the decomposition and release of sulfur-containing species retained within the graphite layers [66]. Elemental analyses of commercially available expanded graphite have likewise demonstrated measurable sulfur contents, with the level depending on the type of intercalant and processing history [67]. Therefore, the detection of SO_2_ in the present composites can reasonably be attributed to the sulfur-containing fraction associated with the expanded graphite rather than to the reinforcing fibers, vermiculite, glass microspheres or ammonium polyphosphate (APP), provided that these constituents are sulfur-free according to their formulations. 

The formation of SO_2_ during heating of sulfur-containing expandable graphite is related to the decomposition of the intercalated species and does not require assuming a specific reduction reaction between sulfuric-acid-derived species and the carbonaceous matrix. In particular, thermal-volatilization experiments have shown that SO_2_ becomes a measurable product at elevated temperatures, following earlier stages of graphite expansion and volatile release [66]. This interpretation is also consistent with recent studies describing sulfuric-acid-intercalated graphite as a source of gaseous products during thermal expansion, including sulfur-containing species [68]. Consequently, the SO_2_ detected in the present fire effluents may be regarded as a characteristic gaseous product associated with the sulfur-containing expanded-graphite component of the hybrid formulations. 

At the same time, the presence of SO_2_ should not be interpreted as evidence of a beneficial dilution effect sufficient to reduce the overall fire hazard. Although non-combustible gaseous products released by flame-retardant systems can modify the composition of the gas phase, their presence may also accompany changes in combustion efficiency and the formation of incomplete-combustion products. Recent studies on flame-retardant epoxy systems have shown that the development of a protective condensed-phase residue can restrict oxygen transport to the underlying polymer, resulting in increased CO formation and a higher contribution of incomplete combustion products [69]. Similar relationships have been reported for other flame-retardant epoxy formulations, in which disruption of gas-phase combustion was associated with increased CO/CO_2_ ratios and changes in smoke production [70]. Thus, the higher concentrations of selected gaseous products observed for the hybrid composites should be interpreted as the combined result of altered heat and mass transfer, condensed-phase protection, flame inhibition and the specific chemical composition of the flame-retardant system, rather than being attributed exclusively to oxygen deficiency.

## 4. Discussion

The results show that the fire behavior of the investigated hybrid epoxy composites depends on the combined effects of the EG/APP flame-retardant system and the specific reinforcement and filler architecture. Although all formulations contained the same general flame-retardant system, substantial differences were observed in ignition resistance, heat release, smoke generation, thermo-oxidative degradation, and gaseous combustion products. This indicates that flame-retardant effectiveness cannot be considered solely a property of the additives; it also depends on their interactions with the overall composite structure.

Cone calorimetry revealed distinct fire-response patterns rather than a uniform improvement across all parameters. Several hybrid formulations showed a clear delay in ignition and in the development of the heat-release process. ACGBF had the lowest average heat release rate (8 kW·m^−2^), MARHE (20 kW·m^−2^), and total heat release (4 MJ·m^−2^), although its pHRR (97 kW·m^−2^) was slightly higher than that of reference G (92 kW·m^−2^). VACMGBHF showed the longest time to ignition (206 s), the latest pHRR (1050 s), a pHRR of 79 kW·m^−2^, and a mass loss of 16%. These findings indicate that ACGBF and VACMGBHF differed in the way heat was released. The former showed very low overall heat release despite a short time to pHRR, whereas the latter delayed ignition and the development of peak heat release. The post-test residues varied in morphology and cohesion, with hybrid composites generally displaying more pronounced carbonaceous–mineral structures than the glass-fabric reference. This is consistent with a role for the composite architecture in shaping the condensed-phase barrier, although the contribution of individual constituents cannot be isolated from the present data. 

Thermal analysis supported the differences observed in the cone calorimeter. During the principal degradation stage, VACMGBHF had the lowest maximum mass-loss rate among the hybrid formulations (2.03%·min^−1^ at 320 °C) and the lowest mass loss in that stage (21.4%). It also retained the highest residual mass at 900 °C among the hybrid composites (38.8%). One plausible explanation is that the specific arrangement and composition of its reinforcing fabrics and fillers, together with the EG/APP system, favored a more gradual decomposition and retention of a larger thermally stable fraction. The multi-layered residue observed after cone calorimetry is consistent with this interpretation. However, these results do not independently demonstrate a particular microscopic char-forming or barrier mechanism. The reference composite G retained the highest residue overall (58.8%), which is consistent with its glass-fabric-only architecture and high inorganic reinforcement fraction. The corresponding STA/FT-IR results also showed a lower intensity of evolved gaseous products during the principal degradation stages. Importantly, the combined TG/DTG, DSC, and FT-IR results provide evidence of this behavior but should not be interpreted as independent proof of a particular microscopic char-forming mechanism.

The exothermic effects observed in the corresponding DSC curves at elevated temperatures support the occurrence of oxidation-related processes. Nevertheless, the simultaneous presence of several organic and inorganic constituents prevents the individual contribution of each component to the observed mass losses from being determined using TG/DTG and DSC alone. Therefore, the high-temperature mass-loss stages should be interpreted as the result of overlapping oxidation and thermal transformation processes involving the composite residue.

The composition of the investigated composites provides additional insight into the differences observed in their thermal behavior and the amount of residue retained at 900 °C. The additive contents were calculated relative to the combined mass of the epoxy resin and hardener and included 25.5% vermiculite, 2.5% glass microspheres, 12.5% hazelnut-shell filler, 10% expandable graphite and 10% ammonium polyphosphate (APP). These values therefore do not represent the mass fractions of the additives in the entire composite. The mineral fillers were not present in all formulations: vermiculite and glass microspheres were incorporated in VCAMGBHF and VACMGBHF, whereas VAGCBHF contained vermiculite but no glass microspheres. Hazelnut-shell filler was used in the formulations containing this organic filler. In contrast to the mineral constituents, hazelnut shells undergo thermal decomposition and may contribute to the formation of carbonaceous residue. Vermiculite, owing to its mineral structure and thermal stability, may remain partly in the solid residue, while glass microspheres may contribute to its inorganic fraction.

The EP/APP flame-retardant system may additionally influence the formation and thermal stability of the condensed-phase residue. Graphite can contribute to the formation of a carbon-rich layer, whereas APP may promote the formation of phosphorus-containing products that can remain in the condensed phase after thermal decomposition. Consequently, the residual mass measured at 900 °C should be regarded as the combined contribution of the glass and basalt reinforcements, mineral fillers, carbonaceous residues and thermally stable products originating from the flame-retardant system. The differences in residual mass between the investigated composites may therefore reflect their different reinforcement and filler architectures, as well as the interactions between these constituents during thermal degradation. Nevertheless, the individual contribution of each component cannot be quantified from the TG/DTG and DSC results alone. Further chemical and elemental characterization of the residues would be required to establish their composition and to determine the contribution of individual constituents more precisely.

An important finding of the study is that improved heat-release characteristics do not necessarily correspond to reduced smoke formation. Most hybrid formulations exhibited higher smoke optical density than the reference composite G, despite their modified heat-release profiles. This confirms that smoke generation represents a separate aspect of fire behavior and is strongly dependent on the composition and structure of the composite. The presence of organic fillers and different fiber types can modify the degradation pathway, volatile release and oxidation efficiency, while the formation of a protective condensed phase can simultaneously alter the transport and combustion of gaseous products. Consequently, assessment based only on HRR or THR would not provide a sufficiently complete description of the fire hazard of these materials.

The analysis of combustion products further demonstrates the complexity of this relationship. The glass-fabric reference composite G generally produced lower concentrations of the selected gaseous products under the applied steady-state conditions, whereas the hybrid formulations showed increased emissions of selected light hydrocarbons and incomplete-combustion products. These differences should be considered in conjunction with the altered heat and mass transfer caused by the formation of the condensed residue. The nitrogen-containing products observed for the hybrid materials are consistent with the presence of nitrogen-containing constituents in the formulations. However, the available measurements do not permit individual formation pathways to be assigned unequivocally. Similarly, the detection of SO_2_ is consistent with the sulfur-containing chemistry associated with the expanded graphite used in the flame-retardant system. These observations emphasize that the introduction of a flame-retardant system can modify not only flame development and heat release but also the composition of fire effluents.

From a material-design perspective, the results indicate that flame retardancy in hybrid polymer composites should be considered a multidimensional property. The investigated formulations demonstrate that delaying ignition, reducing heat release, increasing thermal residue, and limiting smoke generation or hazardous gaseous emissions are related but distinct effects. The common EG/APP system provides the principal flame-retardant functionality, while the reinforcement configuration and filler composition substantially influence how this functionality is expressed in the final composite. The combination of high mechanical strength and improved fire performance makes these composites promising candidates for a broad range of applications in which high strength and fire resistance are required.

All investigated materials achieved the HB classification in the applied horizontal burning test, indicating limited flame propagation under the specific test conditions. However, this classification alone does not fully characterize fire performance under different exposure scenarios. Overall, the findings highlight the importance of a comprehensive approach to evaluating flame-retardant hybrid composites, as no single parameter adequately describes their behavior under fire conditions. The results provide a basis for selecting and further optimizing composite formulations according to the specific mechanical and fire-safety requirements of their intended applications.

## 5. Conclusions

The experimental results revealed differences in the fire behavior, thermal degradation, and smoke production of the investigated composites. VACMGBHF exhibited the longest time to ignition (206 s) and the lowest peak heat release rate (79 kW·m^−2^). In contrast, ACGBF showed the lowest average heat release rate (8 kW·m^−2^), maximum average rate of heat emission (MARHE, 20 kW·m^−2^), and total heat release (4 MJ·m^−2^). Smoke production also varied among the investigated materials. The lowest maximum specific optical density was recorded for the G reference (Ds = 42), while VAGCBHF exhibited the lowest value among the hybrid composites (Ds = 51). ACGBF showed the lowest total smoke release (123 m^2^·m^−2^). Thermal analysis showed that VACMGBHF had the lowest maximum mass-loss rate during the principal degradation stage (2.03%·min^−1^) and the highest residual mass at 900 °C among the hybrid composites (38.8%). The G reference exhibited the highest residual mass overall (58.8%). TG/DTG, DSC, and STA/FT-IR analyses further revealed formulation-dependent differences in degradation stages, thermal effects, and evolved-gas profiles. All investigated composites achieved the HB classification in the horizontal burning test.

Overall, differences were observed among the investigated composites in terms of ignition time, heat release, smoke production, and thermal stability. The results indicate that the measured fire-related properties varied between the tested formulations. However, because the composites differed in several aspects of their composition and reinforcement architecture, the contribution of individual constituents to these differences cannot be determined unambiguously from the present results. No single material exhibited the best performance across all evaluated parameters.

The results provide comparative experimental data on the fire behavior and thermal properties of hybrid epoxy composites containing synthetic and natural reinforcements and functional fillers. Further studies are needed to determine the contribution of individual components and their possible interactions. Such investigations should include systematically designed control samples, together with detailed chemical and morphological characterization of post-test residues. This would help clarify the relationships between composite composition, thermal degradation, and fire behavior.

## 6. Patents

Polish Patent No. PL 248017 B1, entitled “Hybrydowy kompozyt epoksydowy o zmniejszonej palności i dymotwórczości” (“Hybrid epoxy composite with reduced flammability and smoke generation”), was granted in 2025, with protection effective from 2022. The patented solution concerns the development of hybrid composites with reduced flammability and smoke generation, which is an important technological aspect of the present research.

## Figures and Tables

**Table 1:** Composition of prepared and tested composites.

Sample Symbol	A (f. Aramid)	C (f. Carbon)	G (f. Glass)	B (f. Basalt)	L (f. Flax)	V (Vermiculite)	M (Mikrobalon)	H (Hazelnut Shells)
CAGBF	2b	2a	2c	2d	2e	-	-	-
VCAMGBHF	2b	1a	1c	2d	1e	1a	1c	1e
ACGBF	2a	2b	2c	2d	2e	-	-	-
VACMGBHF	1a	2b	1c	2d	1e	1a	1c	1e
VAGCBHF	1a	2c	2b	2d	1e	1a	-	1e
G	-	-	6a	-	-	-	-	-

1–2; 6 refers to the number of layers applied, while “a–e” refers to the order.

**Table 2:** The results obtained from the cone calorimeter test of the tested materials.

Sample Symbol	TTI	TTF	av-HRR	pHRR	t-pHRR	MARHE	THR	TSR	SEA	Mass Loss
	s	s	kW·m^−2^	kW·m^−2^	s	kW·m^−2^	MJ·m^−2^	m^2^·m^−2^	m^2^·kg^−1^	%
CAGBF	127 (16) *	1038 (247)	55 (11)	134 (17)	723 (110)	60 (9)	56 (2)	1026 (208)	311 (50)	29 (2)
VCAMGBHF	186 (39)	1320 (96)	48 (8)	109 (22)	783 (67)	28 (7)	30 (3)	984 (123)	375 (33)	25 (2)
ACGBF	87 (9)	123 (9)	8 (0)	97 (3)	100 (10)	20 (3)	4 (1)	123 (16)	312 (50)	26 (0)
VACMGBHF	206 (20)	1505 (267)	50 (18)	79 (27)	1050 (14)	45 (14)	70 (14)	492 (32)	207 (26)	16 (1)
VAGCBHF	160 (23)	1441 (6)	32 (4)	94 (2)	258 (74)	45 (15)	65 (24)	720 (26)	271 (78)	25 (1)
G	139 (45)	968 (37)	47 (9)	92 (10)	290 (14)	35 (10)	35 (7)	219 (2)	316 (4)	10 (1)

* The values in parentheses are the standard deviations (*n* = 3).

**Table 3:** Results of the smoke density chamber and fire-hazard tests for the investigated materials.

Sample Symbol	Ds	VOF4	Loss of Weight	Classification
	-	-	%	(Horizontal Burning)
CAGBF	142 (51) *	16 (7)	6 (0)	HB
VCAMGBHF	67 (5)	54 (4)	4 (0)	HB
ACGBF	82 (5)	13 (2)	5 (0)	HB
VACMGBHF	57 (1)	43 (3)	4 (1)	HB
VAGCBHF	51 (5)	25 (4)	3 (0)	HB
G	42 (2)	34 (8)	3 (1)	HB

* The values in parentheses are the standard deviations (*n* = 3).

**Table 4:** The results obtained from the thermal analysis of the tested materials.

Sample Symbol	m1	T1	m2	T2	m3	T3	m4	T4	m5	T5	m6	T6	m7	T7	U_900_
	%	°C; %·min^−1^	%	°C; %·min^−1^	%	°C; %·min^−1^	%	°C; %·min^−1^	%	°C; %·min^−1^	%	°C; %·min^−1^	%	°C; %·min^−1^	%
CAGBF	-	-	42.0 (1.2)	332 (1.9); 6.21	-	-	43.6 (1.0)	552 (2.5); 2.41	-	-	-	-	-	-	14.4 (1.5)
VCAMGBHF	-	-	31.6 (1.1)	340 (2.0); 3.74	-	-	43.5 (0.9)	552 (2.7); 2.19	-	-	-	-	-	-	24.9 (1.7)
ACGBF	2.5 (0.1) *	234 (1.5); 0.73	27.6 (0.9)	338 (1.9); 3.27	-	-	26.3 (1.1)	551 (1.9); 2.74	6.6 (0.9)	612 (2.1); 1.54	10.9 (1.1)	780 (2.4); 0.57	-	-	26.2 (1.6)
VACMGBHF	-	-	21.4 (1.9)	320 (1.2); 2.03	-	-	23.2 (0.7)	541 (2.9); 1;71	-	-	8.5 (0.8)	751 (2.6); 0.70	8.1 (1.0)	925 (2.5); 1.56	38.8 (2.0)
VAGCBHF	2.0 (0.1)	251 (1.7); 0.83	32.7 (1.0)	343 (1.3); 4.36	-	-	32.5 (0.8)	536 (3.0); 1.92	-	-	-	-	-	-	32.6 (1.9)
G	-	-	13.0 (0.5)	331 (1.4); 2.10	9.4 (0.6)	356 (1.7); 1.95	18.8 (1.2)	535 (2.3); 1.30	-	-	-	-	-	-	58.8 (1.5)

* The values in parentheses are the standard deviations (*n* = 3).

## Data Availability

The original contributions presented in this study are included in the article. Further inquiries can be directed to the corresponding author.
